# Therapeutic Opportunities for Food Supplements in Neurodegenerative Disease and Depression

**DOI:** 10.3389/fnut.2021.669846

**Published:** 2021-05-14

**Authors:** Rita Businaro, David Vauzour, Jerome Sarris, Gerald Münch, Erika Gyengesi, Laura Brogelli, Pedro Zuzarte

**Affiliations:** ^1^Department of Medico-Surgical Sciences and Biotechnologies, Sapienza University of Rome, Rome, Italy; ^2^Faculty of Medicine and Health Sciences, Norwich Medical School, University of East Anglia, Norwich, United Kingdom; ^3^NICM Health Research Institute, Western Sydney University, Westmead, NSW, Australia; ^4^Professorial Unit, The Melbourne Clinic, Department of Psychiatry, Melbourne University, Melbourne, VIC, Australia; ^5^Pharmacology Unit, School of Medicine, Western Sydney University, Campbelltown, NSW, Australia; ^6^Polistudium srl, Millan, Italy; ^7^Psychiatric Clinic, Faculty of Medicine, University of Lisbon, Lisbon, Portugal; ^8^Neuropsychiatry Research Department, GNR Clinical Center, Lisbon, Portugal

**Keywords:** brain health, depression, neuroinflammation, diet, gut-brain axis

## Abstract

Emerging evidence is showing nutrition as a crucial factor in the high prevalence and incidence of neurodegenerative mental disorders. Preventive interventions on neuroinflammation seem to be able to interfere with neurodegeneration. Supplementation of essential nutrients, such as long-chain-polyunsaturated fatty acids, vitamin E and mineral elements, may minimize inflammation, enhancing antioxidative defense, and lowering the risk and incidence of age-related diseases, such as cardiovascular diseases and neurodegenerative diseases. This manuscript reviews the current evidence on the role of neuroinflammation in the pathophysiology of neurodegenerative and mental disorders, and preventive strategies for food supplementation in these neuropsychiatric diseases. Dietary supplementation-based strategies have been demonstrated to be effective in subjects with mild cognitive impairment, while weaker results have been obtained in patients with advance neurodegenerative disease. Adjunctive supplementation has also been demonstrated to improve depression, this being of marked benefit considering the comorbidity between cognitive impairment/dementia and depression. Further research is needed to improve the prescriptive precision of supplementation in patients, and to better understand potential interactions with clinical and pharmacokinetic factors.

## Introduction

Cognitive impairment is a relevant manifestation of neurodegenerative diseases, which are a heterogeneous group of nervous system conditions, including Alzheimer's disease (AD), Parkinson's disease, Lewy body dementia, and vascular dementia (1). These disorders are characterized pathologically by the abnormal deposition of proteins throughout the brain and spinal cord; glial activation; increased neuroinflammation and changes in metabolic functions of the central and peripheral nervous system (1). Clinical manifestations of these conditions, such as depression and dementia, afflict a growing population worldwide and represent a significant healthcare, social and public policy burden. As an example, over 50 million people live with dementia in the world today and this number is projected to increase to 152 million by 2050 (2). In fact, dementia is considered a mental health issue, with neuropsychiatric symptoms, including depression. The actual prevalence of this latter manifestation can be high in AD patients, with great variability of estimates across studies according to criteria, ranging from 13% (3) to up to 97% (4). Moreover, depression occurring in mid-life or across the lifespan is associated with an increased risk of dementia (5).

Brain vulnerability is influenced by both non-modifiable and modifiable risk factors, including age, family history and genetics on one hand, and preventable cardiovascular risk, obesity, diabetes, sleep apnea, physical activity, tobacco/alcohol/drugs, and stress, on the other hand (6, 7). Diet is one of the most important lifestyle-related factors which may impact on brain vulnerability. Emerging evidence is showing nutrition as a crucial factor in the high prevalence and incidence of mental disorders suggesting that diet is important to brain health as much as to the health of other physiological systems, such as the cardiovascular, endocrine, and digestive systems (8). Micronutrients, such as vitamins and trace mineral elements, are involved in every cellular/biochemical process and play important roles in the brain and heart function, immunological responses, and antioxidant defense systems. Low levels of micronutrients reduce the activity of antioxidant enzymes, which may lead to DNA, protein, and fatty acid oxidation and crosslinking, along with mitochondrial ATP depletion, therefore contributing to cardiovascular or neurodegenerative disorders (9). n-3 Polyunsaturated fatty acids are involved in the maintenance of cognitive functions, promoting adult neurogenesis and neuronal plasticity through the modulation of membrane remodeling, inflammatory mediators and oxidative stress (10).

Growing evidence shows that the release of pro-inflammatory cytokines and mediators in the nervous system is associated with persistent stress stimuli and may lead to neuronal dysfunction and death (11–14). Recent studies also highlight the role of the gut microbiota in many neurodegenerative diseases, as a “microbiota–gut–brain axis” would synchronize the gut with the central nervous system (CNS) and modify the behavior and brain immune homeostasis (15). Modulation of the gut microbiota may be a tractable strategy for developing novel strategies for complex CNS disorders (16). Supplementation of essential nutrients, such as long-chain polyunsaturated fatty acids (LC-PUFAs), vitamin E and mineral elements, may minimize inflammation and enhance antioxidative defense. Consequently, it may lead to lowering the risk and incidence of age-related diseases, including neurodegenerative diseases.

This manuscript reviews the current evidence on the role neuroinflammation in the pathophysiology of neurodegenerative and mental disorders, and nutrient based preventive strategies for food supplementation in these neuro-psychiatric dysfunctions.

## Inflammatory Mechanisms in Neurodegeneration and Depression

Functional and cognitive impairments featuring dementia, in particular AD, vascular dementia and Parkinson's disease, have been associated with depression (17, 18). Concurrent preclinical and clinical evidence has suggested in recent years that in a subset of patients, inflammatory processes and decreased cerebral levels of neurotrophic factors might play some role in the complex pathogenesis of depression (19). Volume reduction of the hippocampus was also observed in AD and depressed patients, and this morphological alteration was related to stress exposure, known to impair the dendritic complexity of the neurons in the CA3 subfield of the hippocampus and affects neurogenesis in the dentate gyrus (20–22). Oxidative stress plays a role in the pathophysiology of depression in bipolar disorder, and high levels of lipid peroxidation in the blood correlates with decreased dentate gyrus volume (23). The notion that depression, as well as infection, encompasses symptoms, such as malaise, anhedonia, decreased social behavior, decreased motor activity, sleep abnormalities and fatigue, which are collectively referred as “sickness behavior,” also suggests the involvement of inflammatory processes in depression development. Such symptoms are confirmed by acute occurrence of depressive symptoms in healthy volunteers administered with lipopolysaccharide (24). Stress syndrome—that is, elevated cortisol levels—has been observed in up to 70% of patients with depression, and also in AD pathology (25).

The role of inflammation in the pathogenesis of depression is supported by studies showing increased levels of proinflammatory cytokines, such as IL-1β, IL-6, IL-12, TNF-α, and prostaglandin E2 (PGE2), in patients with depression (26, 27), and by some experiments in preclinical models in which the administration of inflammatory cytokines promoted a depressive-like syndrome (28, 29). Though it should be noted that inflammation occurs in a sub-set of people with depression and is not always apparent via cytokine blood assays (30). Both depression and heart disease have been linked to the impairment of the cholinergic anti-inflammatory pathway and have been proposed as clinical manifestations of one underlying mechanism (31, 32).

Persistent stress stimuli have, however, been observed to induce the synthesis and the release of pro-inflammatory cytokines and chemokines, such as IL-1β, mainly by neurons and the macrophage–monocyte line, including microglia, resulting in increased expression of inducible nitric oxide synthase and cyclooxygenase. Hydrogen peroxide acts as a cell-to-cell messenger, in the process of inflammatory responses induced by oxidative stress (33). In addition, neurons and glial cells can produce proinflammatory cytokines, such as IL-1β, IL-6, and TNF-α (11, 14, 34, 35).

One further mechanism of depression is a deficit of neurotrophic factors, such as brain-derived neurotrophic factor (BDNF), which leads to altered synaptic plasticity and then to neuronal dysfunction and cell death (18, 36). Inadequate supply of trophic factors also affects neurogenesis, reducing the number of neuronal stem cells able to multiply and differentiate. Several studies on stressed rodents and on patients with depression detected decreased levels of BDNF, together with abnormalities in the levels of neurotransmitters and neuroendocrine dysfunction (18). In humans, it was also reported that BDNF decreases with age and that higher levels of BDNF correlate with better cognitive performance in older adults (36).

### The Role of Microglia

It has been observed that morphological and functional alterations of the microglia appear in adults suffering from depression. Such findings were also replicated in numerous experimental animal models subjected to chronic stress where high levels of proinflammatory cytokines were found to be secreted mainly by activated microglia within the brain. Moreover, an elevated level of microglia activation was detected in individuals with depression who had committed suicide (37).

A microglial signature dependent on TGF-β signaling was demonstrated and was found to be activated by any type of pathologic event or change in brain homeostasis (38). Microglia can strongly influence the pathologic outcome or response to a stressor due to the release of a plethora of substances, including cytokines, chemokines and growth factors. A diversity of microglia phenotypes has been described in response to health, aging, disease, and lifestyle (39). Consequently, the activation of microglia with release of cytokines and neuroinflammation takes place in some individuals and not in others (40). Briefly, catecholamines released during stress bind to receptors expressed by innate immune cells, this binding induces the synthesis and release of inflammatory cytokines that stimulate the production of corticotropin-releasing factor (CRF) by the hypothalamic paraventricular neurons. CRF transported by portal hypophyseal circulation targets the adenohypophysis leading to the release of adrenocorticotropic hormone (ACTH) that binds to adrenal glands to induce cortisol release. Cortisol has a double paradoxical effect: it has anti-inflammatory activity in the periphery and pro-inflammatory activity at the CNS level, where specific receptors are present within the hippocampus and amygdala. Stress conditions, as well as an exogenous application of glucocorticoids, can cause hippocampal neuronal damage and cognitive impairment (41–43).

## Rationale for Food Supplementation to Control Neuroinflammation

Since food is one of the most important lifestyle factors accounting for mental health, one of the strategies currently being developed to treat depression, as well as AD, is to administer compounds with anti-inflammatory and antioxidant activity, capable of crossing the blood–brain barrier, and targeting cells and the molecular players of neuroinflammation. The relevance of this attempt is confirmed by evidence that treatment with anti-inflammatory drugs had beneficial effects on patients with major depression, although some studies failed to demonstrate that COX-2 inhibitors had an activity superior to placebo, most likely due to the fact that COX inhibitors do not affect the expression of pro-inflammatory cytokines and chemokines (44–47). As it is known that natural compounds present in plant-based foods, such as fruits (especially berries), display anti-inflammatory neuroprotective activity, nutritional protocols aiming at counteracting the progression of chronic diseases have been proposed (48–57). Interest has also focused on dietary patterns, such as feeding time and circadian rhythms, to increase availability of bioactive compounds capable of exerting both anti-inflammatory and antioxidant functions. The field of “nutritional psychiatry” aims to describe and understand the relationship between dietary factors and mental health disorders (10, 58).

n-3 LC-PUFAs (a.k.a. omega-3) are precursors of a series of lipid mediators, including resolvins, protectins and maresins, which are collectively termed “specialized pro-resolving mediators” (SPM), and which terminate inflammatory processes blocking polymorphonucleate migration and promoting macrophage M2 switch (59). Several preclinical and clinical studies have shown that n-3 LC-PUFAs are involved in the maintenance of cognitive functions acting on membrane remodeling, inflammation mediators and oxidative stress. They are considered beneficial for the CNS via the modulation of adult neurogenesis, synaptic and neuronal plasticity, and microglia activation (10). Docosahexaenoic acid (DHA) is a key structural component of membrane phospholipids in the brain and eicosapentaenoic acid (EPA) is a precursor of anti-inflammatory cytokines, an inhibitor of prostaglandins, thromboxanes and leukotrienes. In contrast, linoleic acid, a n-6 LC-PUFA (a.k.a. omega-6), is a precursor of the inflammatory mediator arachidonic acid (10). DHA is 250–300-times more abundant in brain tissue compared to EPA (60). It is known that EPA and DHA can cross the blood–brain barrier by diffusion and by blood-brain barrier transporters (61). The latter mechanisms could be defective in aged people or subjects with related genotypes, such as *APOE4*, suggesting higher requirements in population subgroups (62).

DHA intake has been related to lower rates of incident dementia. In populations with higher dietary intake of DHA and higher concentrations of plasma DHA there is a lower risk of cognitive impairment (61). In addition, peripheral blood macrophages obtained from patients with mild cognitive impairment (MCI) and AD, were unable to accomplish an effective phagocytosis for Aβ peptide (the main component of the amyloid plaques in AD), unlike macrophages obtained from healthy controls which could recover phagocytic functions via n-3-LC-PUFA. Recent studies of fish-derived omega-3 supplementation in patients with AD and MCI have shown polarization of *APOE3/E3* patients' macrophages to an intermediate M1–M2 phenotype that is optimal for Aβ peptide phagocytosis and the stabilization of cognitive decline (63, 64).

Moreover, DHA can increase neuronal membrane fluidity and decrease membrane peroxidation by reducing cholesterol levels in the cell membrane, which leads to reduced oxidative stress in the cerebral cortex and in the hippocampus (65, 66). DHA red blood cell content has been related with memory performance, as 2.4 g of EPA plus DHA/day significantly improved red blood cell membrane EPA+DHA composition in older adults with memory impairment, and working memory and neuronal response were concomitantly improved (66). Other clinical trials showed different outcomes of the omega-3, such as EPA, docosapentaenoic acid (DPA), and DHA, supplementation, suggesting its effectiveness in the prevention of dementia although not in the treatment of overt dementia (65); this probably is in relation to the different genotypes of the subjects included in the trial (e.g., carriers of the *E4* allele) (67). Dietary supplementation of EPA, DPA, and DHA in aged rats restored reduced depolarization-induced transmitter release, by changing the membrane composition, while healthy human adults who received EPA supplementation have decreased serum proinflammatory cytokines levels (59, 68).

Great interest was also focused on the properties of some compounds to exert an antioxidant activity, such as vitamin E, and on the ability to interfere with one carbon metabolism by decreasing the quantities of homocysteine, such as the B vitamins (49). Administration of vitamin E at 2,000 IU/day was reported to slow functional decline in mild and mild to moderate AD, while it had no effect on subjects with MCI (69). Moreover, preclinical studies have reinforced the hypothesis of an antidepressant-like response of vitamin E, as the mechanisms underlying its effect seem to be related to the modulation of oxidative stress and neuroinflammation (70).

## Variability in Response to Nutraceuticals Intake: The Role of the Gut–Brain Axis and the Microbiome During Aging and Neurodegenerative Diseases

Although dietary supplements can be obtained and consumed without medical prescription, criteria should be necessary to identify patients affected with neurodegenerative diseases who would most benefit from such an approach. As an example, a meta-analysis of randomized controlled trials (RCT) found that higher intake of the omega-3 EPA and DHA improved specific cognitive domains in subjects with MCI without dementia but had no effects in healthy adults and those with AD (71). This suggests that people in the early stages of progression of cognitive decline and depression may benefit from treatment with omega-3, but that advanced disease is not susceptible to improvement. So, eligible patients need to be identified when such supplementation is proposed in a clinical setting. In this respect, a clinical trial carried out in elderly people with MCI demonstrated beneficial effect of B-vitamin treatment on brain atrophy rate of adults with MCI, only in subjects with high plasma n-3 LC-PUFAs (72). Of note, it has been observed that when the omega-3 fatty acids DHA and EPA concentrations are low, vitamin B treatment has no effect on cognitive decline in MCI, but when omega-3 levels are in the upper normal range, vitamin Bs interact to slow cognitive decline (73).

It is known that gut microbes are a relevant factor changing the effect of dietary supplements on the brain. The exposome represents all exogenous and endogenous environmental exposures of which microbiota, genes and lifestyle environment, may determine the metabolism associated with a specific phenotype. It is known that our microbiome is changing with growing age. Indeed, both cell culture-dependent and -independent studies show that the gut microbiota of older people differs from that of younger adults (74). There is no chronological threshold or age at which the composition of the microbiota suddenly alters; rather, changes occur gradually with time. O'Toole and Jeffery (75) showed that the gut microbiota of older people differs from that of younger adults and may modulate aging-related changes in innate immunity, sarcopenia and cognitive functions.

Accumulating evidence now indicates that the gut microbiota also communicates with the CNS, possibly through neural, endocrine and immune pathways, and thereby influences brain function and behavior (11). Thus, the emerging concept of a microbiota–gut–brain axis suggests that modulation of the gut microbiota may be a tractable strategy for developing novel therapeutics for complex CNS disorders (16).

The Human Microbiome Project and subsequent studies using next-generation sequencing technology have highlighted that thousands of different microbial species are present in the human gut, and that there has been a significant variability of taxa in the microbiota composition among people. Several factors (gestational age, mode of delivery, diet, sanitation, and antibiotic treatment) influence the bacterial community in the human gastrointestinal tract, and among these, dietary components, such as n-3 fatty acids, fibers and polyphenols, play a crucial role (76, 77).

A bidirectional communication between the gut and the brain occurs via the immune system, the vagus nerve, the enteric nervous system and microbial metabolites (78). Substances, including short-chain fatty acids (SCFAs), proteins and tryptophan metabolites, exchanged through the circulatory system, affect mood, cognition and other brain function parameters (15, 16).

It is important to consider that gut microbes can produce neurotransmitters, such as dopamine, 5-HT, GABA, and acetylcholine (16). These neurotransmitters may signal to the brain via the vagus nerve. In particular, gut microbes can stimulate immune cells to produce cytokines. These cytokines might target the brain via blood vessels of the circulatory system. In addition, gut microbes can produce metabolites, such as microbial fermentation end-products (SCFAs, e.g., butyrate) that can regulate the epigenetic synthesis of BDNF, for example (79, 80). These metabolites can potentially migrate to the brain via the blood vessels or may stimulate gut epithelial cells to produce neurotransmitters that activate the vagus nerve (16).

Activity of the gut–brain axis seems to be linked to certain genetic variations. One example is the apolipoprotein E4 (*APOE4*) genotype which has been associated with early cognitive decline and is the strongest prevalent genetic risk factor for AD. Apolipoprotein E is a transporter of cholesterol and it is present in the liver (80–90%), in the brain glia and macrophages. It was found to influence the structure and the function of the gut microbiome both in humans and mice (81, 82). This suggests that the gut–brain axis is a potential target to reduce the impact of the *APOE4* allele on cognitive decline and for the prevention of AD (81). For example, fecal microbiota amplicon sequencing from age- and BMI-matched individuals revealed higher levels of *Prevotellaceae* in *APOE3/E3* carriers relative to other genotype subgroups, whereas higher levels of *Ruminococcaceae* were correlated with the *APOE2/E3* genotype relative to *APOE4* carriers. *Ruminococcaceae* are involved in the production of SCFAs, such that their depletion is causally linked to inflammation (83). These findings therefore suggest that these bacteria might contribute to the protective effects of *APOE2* and *APOE3* alleles against AD relative to the *APOE4* genotype (81).

As a confirmation of these concepts, it has been observed that fish oil intake works better in non-*APOE4* carriers. Huang et al. (84) highlighted that consumption of fatty fish was associated with a reduced risk of dementia and AD for those without the *APOE4* allele. Quinn et al. (85) performed a randomized, double-blind, placebo-controlled trial to determine that supplementation with DHA slows cognitive and functional decline in individuals with AD. Participants were randomly assigned to algal DHA at a dose of 2 g/day or to placebo for a duration of treatment of 18 months. Overall, supplementation with DHA compared with placebo did not slow the rate of cognitive and functional decline in patients with mild to moderate Alzheimer disease, but while there was no DHA treatment effect on any outcome measure in the *APOE4*-positive group, those receiving DHA in the *APOE4*-negative group had a significantly lower decline in mean change in Alzheimer's Disease Assessment Scale-Cognitive Subscale score over 18 months vs. placebo group.

More recently, Arellanes et al. (86) demonstrated that supplementation with DHA doses higher than 1 g per day may contribute to reducing certain biomarkers associated with dementia prevention. High doses of DHA are needed for adequate brain bioavailability and *APOE4* is associated with reduced delivery of DHA and EPA to the brain before the onset of cognitive impairment. A total of 33 individuals were provided with two vitamin B complex supplements per day (each containing 500 μg of vitamin B12, 50 mg of vitamin B6, and 400 μg of folic acid) and randomized to 2,152 mg of DHA per day or placebo over 6 months. There was a significant increase in cerebrospinal fluid (CSF) DHA in the DHA supplement arm compared with placebo. It was also observed that a trend for a greater increase in CSF DHA in *APOE4* non-carriers compared with carriers occurred (but the interaction between the treatment group and *APOE* group on the change of CSF DHA was not significant). CSF EPA levels were increased after DHA supplementation, with such results being in line with previous reports (87). However, change in CSF EPA levels in both groups was significantly greater in *APOE4* non-carriers compared to carriers.

As mentioned earlier, EPA and DHA are able to produce SPMs such as RvE1, RvE2, and RvD1, RvD2, respectively, that are involved in the resolution of inflammation (88). Martinsen et al. quantified cortical and hippocampal fatty acid, and phospholipid profiles along with select EPA- and DHA-derived SPMs in 2-, 9- and 18-month-old *APOE3* and *APOE4* male and female mice. A 10% lower cortical DHA was evident in *APOE4* females at 18 mo compared with 2 mo, with no significant decrease in *APOE3* or *APOE4* males. This decrease was associated with a reduction in DHA-phosphatidylethanolamine. In addition, although no sex^*^APOE genotype interactions were observed for SPMs expressed as a ratio of their parent compound, higher cortical RvD3, neuroprotectin D1 (NPD1), maresin 1 (MaR1) were evident in females, and lower cortical 17R-resolvin D1, 10S, 17S-diHDHA, and 18-HEPE in *APOE4* (88).

Presented evidence suggests that precision medicine represents a future perspective for treatment and prevention of neurodegenerative diseases. Genetics, nutrigenetics, and pharmacogenetics have a major role in health maintenance and treatment of diseases. In particular, a genomics, transcriptomics, and epigenomics approach could be useful, and the potential advantages of a genotype-based personalized nutrition could facilitate early, personalized therapy, and improve motivation.

## Therapeutic Opportunities for Food Supplements in Depression: Evidence, Limitations and Suggestions for the Future

Evidence for the potential efficacy of several supplementation strategies in the prevention and treatment of neurodegenerative diseases has been obtained in clinical trials, although inconsistent results were produced. A systematic overview of all available top-tier meta-analyses of RCTs reported on the efficacy of nutrient supplements in patients with common and severe mental disorders (89). In particular, 33 unique meta-analyses with outcome data from placebo-controlled trials were included from a total of 10,951 participants. Results highlighted that omega-3 LC-PUFA, EPA at dose up to 4,400 mg/day (on average 1–2 g of EPA per day) in particular, is significantly effective in depression. Moreover, good evidence supported the effectiveness of high dose of methyl folate (15 mg/day) compared with folic acid and zinc as an adjunctive treatment in major depressive disorder (MDD) (89).

Nevertheless, some clinical trials failed to demonstrate the efficacy of LC-PUFA, either alone or associated with pharmacological or behavioral interventions, in patients with depression (90–93).

Unpromising results were also obtained by some authors researching the impact of the impact of LC-PUFA supplementation on cognitive functions (94–96). In some instances, clinical trials tested complex interventions and obtained data which may be difficult to interpret. As an example, Chew et al. evaluated the effects on cognitive function of oral supplementation with 1 g LC-PUFA and/or lutein 10 mg/zeaxanthin 2 mg in elderly subjects with age-related macular degeneration and failed to demonstrate efficacy of supplementation (97).

It is possible that multinutrient combinations may provide some advantage, with possible favorable synergistic interactions between components. For example, a recent 36-month, double-blind, placebo-controlled study in 311 participants with prodromal AD reported that a multinutrient intervention containing DHA and EPA slowed decline of cognition, function, brain atrophy, and disease progression (98).

It has been observed that adjunctive use of nutraceuticals has the potential to modulate several key neurochemical pathways and can be used as an augmentation strategy to improve inadequate response to antidepressants. In this application, a systematic review and meta-analysis found primarily positive results for replicated studies testing adjunctive S-adenosyl methionine (SAMe), methyl folate, n-3 LC-PUFAs (EPA or ethyl-EPA), and vitamin D; positive isolated studies found efficacy of creatine and an amino acid formula; mixed results were found for zinc, folic acid, and vitamin C; and negative results were found for inositol (99).

Folate deficiency has been reported in approximately one-third of people suffering from depressive disorders (99). A combined folate, B12 and B6 dietary deficiency, induces hyperhomocysteinemia and imbalance of S-adenosylmethionine and S-adenosylhomocysteine, leading to an up-regulation of presenilin1 (PS1) and beta-secretase (BACE) and amyloid beta deposition, promoting progression to AD (100). Several studies tested folic acid adjunctively with antidepressants and most of these studies yielded positive results in regard to enhancing either antidepressant response rates or increasing response onset. The 5-methlytetrahydrofolate (dosage 400 μg to 15 mg) or folinic acid (400–800 μg) are considered safe and potentially effective forms (99).

Available data regarding the adjunctive use of n-3 LC-PUFAs in depression are in favor of n-3 compared with placebo. A recent network meta-analysis of 10 clinical trials including 910 patients, demonstrated that adjuvant supplementation with n-3 PUFAs was superior on MDD symptoms in comparison with placebo, and that high dose n-3 PUFAs (SMD: 0.908 ± 0.331; 95% CI: 0.262–1.581) were more effective than low dose n-3 PUFAs (SMD: 0.601 ± 0.286; 95% CI: 0.034–1.18) (101).

A recent key study, Mischoulon et al. (102), evaluated 196 patients with MDD who were administered with EPA 1 g/day or DHA 1 g/day or placebo for 8 weeks. Significant improvement in depression symptoms as measured by the 17 -item Hamilton Rating Scale (HAM-D-17), the Quick Inventory of Depressive Symptomatology–Self-report (QIDS-SR) and the Clinical Global Impression—Severity scale (CGI-S) was observed but neither active treatment reached statistical significance compared to placebo, with the response rates being 40–50% for each arm with a remission of ~30%. The authors of the study concluded that neither EPA-enriched nor DHA-enriched n-3 was superior to placebo for the treatment of MDD, hypothesizing that baseline levels of inflammatory and metabolic markers, such as human C-reactive protein, IL-6, IL-1RA, leptin, and adiponectin could have an impact on response (102).

Rapaport et al. (103) explored in a *post-hoc* analysis of the above study, whether inflammatory biomarkers act as moderators of clinical response to omega-3 fatty acids in subjects with MDD. It has been observed that, overall, although treatment group differences were negligible (standardized treatment effect size, ES: −0.13–0.04), subjects with “high” inflammation improved more on EPA than placebo (ES: −0.39) or DHA (ES: −0.60) and less on DHA than placebo (ES: 0.21). Furthermore, difference between EPA and placebo effect increased with increasing numbers of markers of high inflammation. Therefore, the authors concluded that employing multiple markers of inflammation facilitated the identification of a more homogeneous cohort of subjects with MDD responding to EPA with an advantage over placebo.

A *post-hoc* analysis of an 8-week, double-blind, RCT (*n* = 158) investigated a combination of nutraceuticals comprising omega-3 (EPA 1 g/DHA 656 mg), SAMe, zinc, 5-hydroxytryptophan, folinic acid, and co-factors vs. placebo for the treatment of MDD. The study explored levels of PUFAs, folate, vitamin B12, zinc, homocysteine and BDNF as possible predictors and correlates of response to nutraceutical supplementation. It has been demonstrated that concentrations of EPA and DHA in red cell membranes increased in response to treatment and were significantly correlated with a decrease in depressive symptoms during active treatment (*p* = 0.003 and *p* = 0.029, respectively). Higher baseline levels of omega-3 fatty acid also correlated with depression reduction in the active treatment group (*p* = 0.011) (99). Therefore, changes in fatty acid levels resulting from a nutraceutical combination containing EPA and DHA provided a biomarker response in treating depression (99).

A subcommittee of the International Society for Nutritional Psychiatry Research organized an expert panel and developed a consensus-based practice guideline for clinical use of n-3 LC-PUFAs in MDD (104). Although a link between low omega-3 levels and depressive symptoms was acknowledged, evidence for supplementation as a prevention strategy was not sufficient. Stronger evidence was found for adjunctive supplementation with antidepressants, and acute use in the presence of obesity or inflammation was deemed as a potentially more effective treatment strategy. Evidence for efficacy of omega-3 supplementation in bipolar disorder was present, while evidence for use in children or elderly was not strong. Application in perinatal usage was based on weak evidence but omega-3 were indicated as safer than antidepressants (105). With respect to formulation and dosage, both pure EPA or an EPA/DHA combination of a ratio higher than 2 are considered effective, and the recommended dosages should be 1–2 g of net EPA daily, from either pure EPA or an EPA/DHA (>2:1) formula.

Current evidence most strongly supports a positive association between zinc deficiency and the risk of depression (106–108). Conversely, the relationship between magnesium and selenium deficiency and depression has not been fully understood. Several hypotheses have been advanced, and selenium and magnesium seem to be involved in the regulation of the hypothalamic–pituitary–adrenal axis, and of glutamate (109).

Owen et al. (110) measured plasma vitamin E (α-tocopherol) in 49 adults with major depression. It has been observed that subjects had significantly lower plasma vitamin E (4.71 ± 0.13 μmol/mmol cholesterol) than has previously been reported for healthy patients, and plasma vitamin E was inversely correlated to depression score. In addition, alpha tocopherol was found to be beneficial in mild to moderate AD by slowing decline of cognition (69).

In conclusion, good evidence supports the efficacy of adjunctive supplementation with EPA, vitamin D, and methyl folate in patients with depression, and mixed results are available for zinc and vitamin C.

## Conclusion

Neuroinflammatory mechanisms based on glial cell inflammatory processes and resulting neuronal dysfunction and impaired neurogenesis have been identified (106). Several neurodegenerative conditions have been linked to persistent oxidative stress, and pathological changes dependent on release of inflammatory mediators. In addition, the role of some nutrients, namely DHA and EPA among others we have not reviewed, in the regulation of microinflammation and central neurotransmission has been elucidated. Therefore, great interest has focused on diet and eating habits which may provide suitable levels of bioactive compounds capable of exerting both anti-inflammatory and antioxidant functions, and their therapeutic potential to prevent neurodegenerative diseases and one of their prodromes, clinical depression.

Dietary supplementation-based strategies have been demonstrated to be effective in people with depression and in those with MCI, while weaker results have been obtained in patients with advanced neurodegenerative disease.

When addressing prevention and treatment of neuroinflammation-dependent conditions by dietary supplementation, beyond the identification of required nutrients, it is necessary to evaluate substance metabolism, absorption and distribution, namely into the CNS, and the interaction with subject's feature including genetic variation and microbiota. The emerging concept of a microbiota–gut–brain axis suggests that modulation of the gut microbiota may be a tractable strategy for developing novel therapeutics for complex CNS disorders, and dietary habits play a crucial role in the selection of the bacterial community in the human gastrointestinal tract (77).

Future research ideally should focus on precision-based approaches tailoring supplementation based on nutrient deficiencies, neurochemical abnormalities or pharmacogenetic differences.

## Author Contributions

All authors listed have made a substantial, direct and intellectual contribution to the work, and approved it for publication.

## Conflict of Interest

JS has received either presentation honoraria, travel support, clinical trial grants, book royalties, or independent consultancy payments from: Integria Healthcare & MediHerb, Pfizer, Scius Health, Key Pharmaceuticals, Taki Mai, Fiji Kava, FIT-BioCeuticals, Blackmores, Soho-Flordis, Healthworld, HealthEd, HealthMasters, Kantar Consulting, Angelini Pharmaceuticals, Grunbiotics, Polistudium, Australian Natural Therapeutics Group, Research Reviews, Elsevier, Chaminade University, International Society for Affective Disorders, Complementary Medicines Australia, SPRIM, Terry White Chemists, ANS, Society for Medicinal Plant and Natural Product Research, Sanofi-Aventis, Omega-3 Center, the National Health and Medical Research Council, CR Roper Fellowship. EG has received grants or PhD fellowship support from Indena Italy SpA and Verdure Sciences. LB is employed in Polistudium, Italy, which received funds for editorial assistance from Angelini. GM has received either presentation honoraria, travel support, grants, or independent consultancy payments from Polistudium, Integria Healthcare, Qbiotics, Soho-Floris, and Pharmako Ltd. The remaining authors declare that the research was conducted in the absence of any commercial or financial relationships that could be construed as a potential conflict of interest.
